# *Nonomuraea corallina* sp. nov., isolated from coastal sediment in Samila Beach, Thailand: insights into secondary metabolite synthesis as anticancer potential

**DOI:** 10.3389/fmicb.2023.1226945

**Published:** 2023-11-20

**Authors:** Chananan Ngamcharungchit, Atsuko Matsumoto, Chanwit Suriyachadkun, Watanalai Panbangred, Yuki Inahashi, Bungonsiri Intra

**Affiliations:** ^1^Department of Biotechnology, Faculty of Science, Mahidol University, Bangkok, Thailand; ^2^Mahidol University and Osaka Collaborative Research Center on Bioscience and Biotechnology, Bangkok, Thailand; ^3^Graduate School of Infection Control Sciences, Kitasato University, Tokyo, Japan; ^4^Kitasato Institute for Life Sciences (O̅mura Satoshi Memorial Institute), Kitasato University, Tokyo, Japan; ^5^Thailand Bioresource Research Center (TBRC), National Science and Technology Development Agency, Pathumthani, Thailand; ^6^Research, Innovation and Partnerships Office – RIPO (Office of the President), King Mongkut’s University of Technology Thonburi, Bangkok, Thailand

**Keywords:** actinomycetes, novel marine taxa, phylogenetic analysis, *Nonomuraea*, anti-colorectal cancer

## Abstract

A novel marine actinomycete, designated strain MCN248^T^, was isolated from the coastal sediment in Songkhla Province, Thailand. Based on the 16S rRNA gene sequences, the new isolate was closely related to *Nonomuraea harbinensis* DSM45887^T^ (99.2%) and *Nonomuraea ferruginea* DSM43553^T^ (98.6%). Phylogenetic analyzes based on the 16S rRNA gene sequences showed that strain MCN248^T^ was clustered with *Nonomuraea harbinensis* DSM45887^T^ and *Nonomuraea ferruginea* DSM43553^T^. However, the digital DNA–DNA hybridization analyzes presented a low relatedness of 40.2% between strain MCN248^T^ and the above closely related strains. This strain contained meso-diaminopimelic acid. The acyl type of the peptidoglycan was acetyl, and mycolic acids were absent. The major menaquinones were MK-9(H_2_) and MK-9(H_4_). The whole cell sugars consisted of madurose, ribose, mannose, and glucose. Diphosphatidylglycerol, hydroxyl-phosphatidylethanolamine, phosphatidylethanolamine, phosphatidylinositol, and phosphatidylglycerol were detected as the major phospholipids. The predominant cellular fatty acids were *iso*-C_16:0_ (40.4%), 10-methyl-C_17:0_ (22.1%), and C_17:1_ ω 8c (10.9%). The DNA G + C content of the genomic DNA was 71.7%. With *in silico* analyzes, the antiSMASH platform uncovered a diverse 29 secondary metabolite biosynthesis arsenal, including non-ribosomal peptide synthetase (NRPS) and polyketide synthase (PKS) of strain MCN248^T^, with a high prevalence of gene cluster encoding pathways for the production of anticancer and cytotoxic compounds. Consistently, the crude extract could inhibit colorectal HCT-116 cancer cells at a final concentration of 50 μg/mL. Based on the polyphasic approach, strain MCN248 was designated as a novel species of the genus *Nonomuraea*, for which the name *Nonomuraea corallina* sp. nov. is proposed. The type strain of the type species is MCN248^T^ (=NBRC115966^T^ = TBRC17110^T^).

## Graphical abstract

“Created with BioRender.com.”

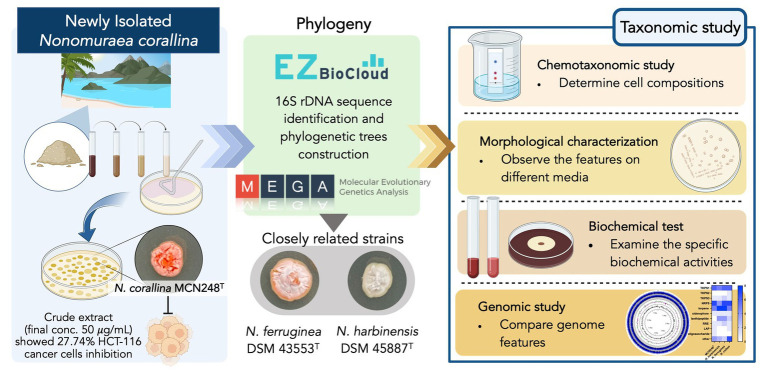

## Introduction

Marine habitats have proven to be significant reservoirs of rare actinomycetes, including the genus *Nonomuraea*, in recent decades. These marine rare actinomycetes are renowned bioactive compound producers, since a total of 267 novel compounds were reported to be produced by them between 2007 and 2017 (Subramani and Sipkema, 2019). The genus *Nonomuraea* produces diverse bioactivity of secondary metabolites; for example, the antibacterial compound actinotiocin was first isolated from *Nonomuraea pusilla* IFO 14684^T^ in 1973 (Tamura et al., 1973). In addition, anticancer, antipsychotic, and biocatalytic compounds, as well as pigments, have been found to be produced by *Nonomuraea* spp. (Sungthong and Nakaew, 2015).

The genus *Nonomuraea* was included in the family Streptosporangiaceae by Quintana et al. (2003). The description of the family was subsequently emended by Stackebrandt et al. (1997), Zhi et al. (2009), Nouioui et al. (2018), and Ay et al. (2020), on the basis of the 16S rRNA gene sequence analysis and chemotaxonomic characteristics. At the time of writing this article, the genus *Nonomuraea* comprises 66 species with validly published names (LPSN, http://www.bacterio.net/nonomuraea.html). These species can be distinguished using a combination of chemotaxonomic, genomic, morphological, and phylogenetic criteria (Lechevalier and Lechevalier, 1970; Embley et al., 1998). They generally produce extensively branching aerial and substrate mycelia. Chains of aerial spores can be hooked, spiral, or straight, and the cell wall is composed of meso-diaminopimelic acid (meso-DAP) (Goodfellow et al., 1990). The major menaquinones are MK-9(H_4_), MK-9(H_2_), and MK-9(H_0_), while the major phospholipids are diphosphatidylglycerol, phosphatidylethanolamine, hydroxylated phosphatidylethanolamine, and ninhydrin-positive phosphoglycolipids. The genomic DNA contains 64.0–73.0 mol% of G + C content (Kämpfer, 2012). The polyphasic approach combines various data sources, including morphological characteristics, DNA sequences, and ecological niches, to enhance the precision of species identification and delimitation (Yang et al., 2022). However, genomics has become a promising methodology as it provides a reproducible, reliable, highly informative means to infer phylogenetic relationships among prokaryotes, which allows the continuation of our tradition toward natural classification (Chun et al., 2018). In this study, we aimed to determine the taxonomic position of an actinomycete isolate that is a novel species of the genus *Nonomuraea* by using a polyphasic taxonomy.

Cancer possesses a significant global challenge to life expectancy. The worldwide cancer burden is projected to increase by 47% to 28.4 million cases by 2040, with transitioning countries facing an even greater increase, ranging from 64 to 95% (Sung et al., 2021). Anticancer drugs undergo metabolism through phase I and phase II metabolizing enzymes, involving oxidation/hydroxylation and hydrolysis (Muhamad and Na-Bangchang, 2020). For instance, curcumin regulates various cellular signaling pathways, including apoptosis, inhibiting progression, and blocking angiogenesis in pancreatic cancer (Kumar et al., 2023). Resistomycin inhibits Pellino-1, an E3 ubiquitin ligase that is reported to have an important role for lymphoid and several solid tumorigeneses, and the inhibition also leads to the downregulation of expression of transcription factors, SNAIL and SLUG, that contribute to tumor weight and lung metastasis in MDA-MB-231 cells (Liu et al., 2020).

## Materials and methods

### Isolation of actinomycetes and 2D anti-cancer screening

Strain MCN248^T^ was isolated from coastal sediment from Samila Beach, Songkhla Province, Thailand (GPS data 7.192455, 100.590469), using a dilution plating technique. A 10-fold dilution of sediment suspension was spread onto starch casein agar (g/L: soluble starch, 10; casein, 0.3; KNO_3_, 2; MgSO_4_.7H_2_O, 0.05; K_2_HPO_4_, 2; NaCl, 2; CaCO_3_, 0.02; FeSO_4_.7H_2_O, 0.01; and agar, 18) supplemented with cycloheximide (50 mg ml^-1^) and nalidixic acid (50 mg ml^-1^) (Wattanasuepsin et al., 2017). The colonies were picked up after incubation for 6 weeks at 28°C. The pure culture was maintained on yeast extract–malt extract agar (International *Streptomyces* Project, ISP 2 medium) at 28°C and stored in 20% (v/v) glycerol at −80°C for long-term preservation.

For the 2D anti-colorectal cancer assay, the crude extract cultivated in A-3 M production medium (g/L: soluble starch, 20; glycerol, 20; pharmamedia, 15; dianion HP-20, 10; glucose, 5; and yeast extract, 3, pH 7) was prepared using ethanol/ethyl acetate extraction and then diluted in DMSO to prepare a stock concentration of 10 mg/mL. To conduct testing against cancer cell line, the crude extract was added to cell plates to obtain a final concentration of 50 μg/mL. High-throughput liquid handling and high-throughput detection system were used for screening compounds through the 3-(4, 5-dimethyl-thiazol-2-yl)-2, 5-diphenyl tetrazolium bromide (MTT) (colorimetric assay) method. The absorbance of this colored solution was measured at a wavelength of 570 nm by a Multi-Mode Microplate Reader (ENVISION). The optical density (OD) was used to calculate the percentage of cell inhibition.

### 16S rRNA sequence identification

PCR amplification was performed using a Perkin Elmer GeneAmp 2400 PCR system and a pair of 11F′ (5′-AGTTTG ATCATGGCTCAG-3′) and 1540R′ (5′-AAGGAGGTGATCCA ACCGCA-3′) universal primers. Sequencing of the 16S rRNA gene of strain MCN248^T^ was carried out by Macrogen, Inc, Korea. The values of sequence similarities among the most closely related strains were computed using the EzBioCloud server (Yoon et al., 2017a,b).^1^ A nearly complete 16S rRNA gene sequence (1471 bp) of strain MCN248T was aligned with multiple sequences of available type strains in the genus *Nonomuraea* on the EZBioCloud database. Phylogenetic trees were constructed using neighbor-joining (Saitou and Nei, 1987) and maximum-likelihood (Felsenstein, 1981) tree-making algorithms in the software package MEGA (version 11) (Tamura et al., 2021). Evolutionary distance matrices were generated according to Kimura’s two-parameter model (Kimura, 1980). The robustness of the tree topologies was assessed by performing bootstrap analysis with 1,000 replicates (Felsenstein, 1985).

### Draft genomic sequencing and *in silico* analyzes

The extraction and purification of chromosomal DNA for DNA G + C content analysis were performed according to the method by Saito and Miura (1963). The draft genome of strain MCN248^T^ was sequenced using the paired-end method and the Illumina HiSeq platform. The G + C content of the genomic DNA of strain MCN248^T^ was calculated from the draft genome sequences. The genomic DNA sequence of *Nonomuraea harbinensis* DSM45887^T^ (GenBank accession no. JAHKRN000000000.1) was obtained from the NCBI database, while that of *Nonomuraea ferruginea* DSM43553^T^ (GenBank accession no. JAPNUD000000000) was first deposited at NCBI in this study. According to the phylogenomic tree construction (Meier-Kolthoff and Göker, 2019), based on genome data, a TYGS-genome blast distance phylogeny (GBDP) was generated using MCN248T and all the available genome data of *Nonomuraea* type strains in the TYGS database. The genomic similarities between strain MCN248^T^ and the above closely related strains were investigated using the average nucleotide identity (ANI) algorithm with the OrthoANIu tool from EZBioCloud software (Yoon et al., 2017a,b) and JSpeciesWS online services (Richter et al., 2015). Additionally, digital DDH (dDDH) analysis was conducted using the Genome-to-Genome Distance Calculator (GGDC) 2.1 platform (Meier-Kolthoff et al., 2013). Analysis of secondary metabolite biosynthesis gene clusters for rapid genome-wide identification was carried out using antiSMASH version 7.0.0beta1 (Medema et al., 2011).

### Chemotaxonomic characterization

Biomass for chemical and molecular studies was harvested by centrifugation after cultivation in tryptic soy broth (TSB) (g/L: tryptone, 17; soytone, 3; sodium chloride, 5; dipotassium phosphate, 2.5; and glucose, 2.5, pH 7.3) at 28°C for 5 days. The purified cell wall was prepared according to the procedure described by Také et al. (2016). The whole-cell sugar composition was examined on cellulose plates using the TLC technique, which was performed following the procedures described by Staneck and Roberts (1974). The presence of mycolic acid was investigated using TLC according to the method by Tomiyasu (1982). Isoprenoid quinones were extracted and subsequently analyzed by liquid chromatography/mass spectrometry (JMS-T100LP, JEOL) with a CAPCELL PAK C18 UG120 column (OSAKA SODA) using methanol/isopropanol (7,3, v/v) and UV detection at 270 nm (Collins et al., 1977; Meyer, 1979; Kroppenstedt et al., 1990; Wang et al., 2014). Phospholipids in cells were extracted and analyzed by two-dimensional TLC according to the method by Minnikin et al. (1977). Methyl esters of cellular fatty acids were prepared by direct transmethylation with methanolic hydrochloride using cells grown in TSB broth for 7 days at 28°C. Cellular fatty acid compositions were identified using a GLC system (HP 6890; Hewlett Packard) assisted by the ACTIN 6 database, according to the instructions for the Sherlock Microbial Identification System (Microbial ID; MIDI, version 6.0) (Kämpfer and Kroppenstedt, 1996).

### Phenotypic characterization

Cultural characteristics were observed after cultivation at 28°C for 14–21 days on International *Streptomyces* Project (ISP) media 2, 3, 4, 5, 6, and 7 (Difco or Nihon Pharmaceutical) (Shirling and Gottlieb, 1966). To specify the colors of aerial and substrate mycelia and diffusible pigment, the Color Harmony Manual was used for color designation (Jacobson et al., 1958). Morphological characteristics of strain MCN248 were observed using scanning electron microscopy (model JSM-5610, JEOL) after incubation on ISP 3 at 28°C for 21 days. Spore-producing samples of the strain were prepared according to previously described methods (Intra et al., 2013).

The temperature range (4–50°C) (temperature gradient incubator TN-2148, Advantec) and pH range (pH 3.0–11.0, at 1 pH unit intervals) for growth and salinity (NaCl) tolerance [0–5% (w/v)] were examined on ISP 2 basal medium after 14 days of incubation. The carbon-source utilization was determined using the methods described by Shirling and Gottlieb (1966) and Williams et al. (1989). The capacity of the strain for starch hydrolysis, hydrogen sulfide production, melanin production, nitrate reduction, gelatin degradation, and casein hydrolysis was investigated using the following media: ISP 4, ISP 6, ISP 7, ISP 8 (0.5% peptone, 0.3% beef extract, and 0.5% KNO_3_, pH 7.0), peptone-glucose-gelatin (2.0% glucose, 0.5% peptone, and 20% gelatin, pH 7.0), and skim milk agar (10% skim milk (Difco) and 1.5% agar, pH 7.0). For the coagulation and peptonization of milk, 10% skim milk (Difco) was used (Kovacs, 1956). A commercial kit API ZYM system (BioMérieux) was used to determine the enzymatic features of the strain according to the manufacturer’s instructions.

## Results and discussion

### Phylogeny of Strain MCN248^T^

**Figure 1:**
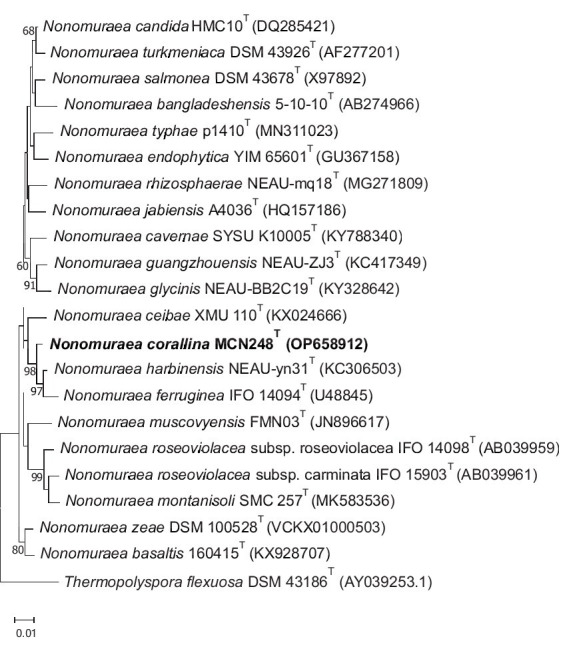
Neighbor-joining phylogenetic tree derived from 16S rRNA nucleotide sequences displaying the genetic relatedness between the MCN248^T^ isolate and other strains in the genus *Nonomuraea*, with *Thermopolyspora flexuosa* as the outgroup. Bootstrap values of 50% or higher are indicated at the branch points (percentages are based on 1,000 resamplings). The scale bar represents 0.01 nucleotide substitutions per site.

Based on pairwise comparison of the almost-complete 16S rRNA gene sequences, strain MCN248^T^ displayed a close association between members of the genus *Nonomuraea.* The highest similarity value to strain MCN248^T^ was *N. harbinensis* DSM45887^T^ (99.2%). Congruent with these results, the neighbor-joining phylogenetic tree indicated that the strain MCN248^T^ was positioned within the genus *Nonomuraea*, where it formed a clade with *N. harbinensis* DSM45887^T^ and *N. ferruginea* DSM43553^T^ (Figure 1). The phylogenetic relationships of 40 *Nonomuraea* species were also supported by the neighbor-joining and maximum-likelihood trees, as shown in Supplementary Figures S1, S2, respectively.

### Draft genomic characterization and *in silico* secondary metabolite cluster profiles

**Figure 2:**
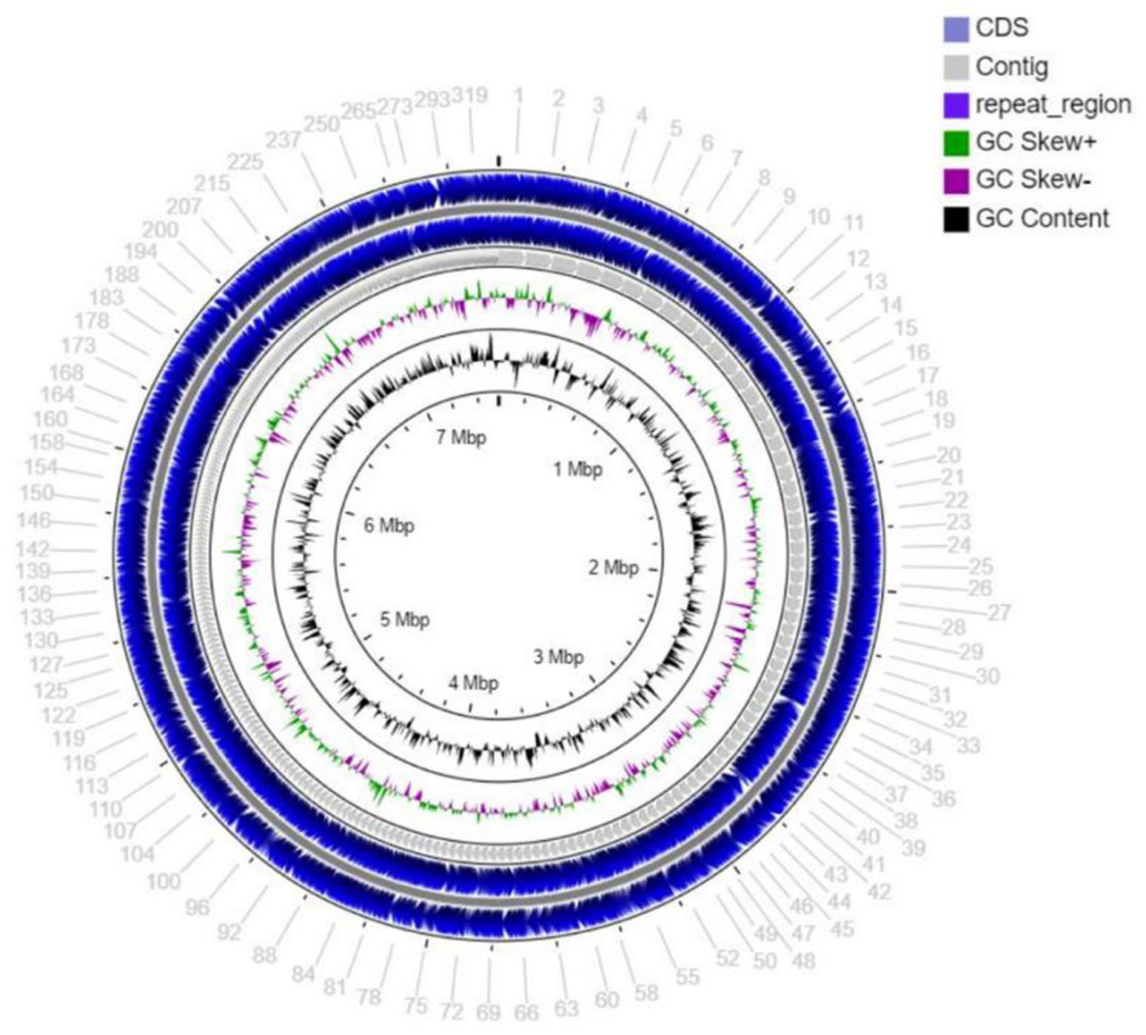
Circular map of the total length 7,557,982 bp draft genome visualized by CG viewer server, showing coding sequence (CDS), contig, repeat region, GC skew+, GC skew-, and GC content of stain MCN248.

The draft genome sequencing of strain MCN248^T^ yielded a genome of 7,557,982 bp (Figure 2) in length after assembly, which produced 370 contigs with an N50 value of 34,089 bp. The G + C content of strain MCN248^T^, calculated from the draft genome sequence, was determined to be 71.7 mol% (Supplementary Table S1).

The dDDH value for comparing strain MCN248^T^ with both *N. harbinensis* DSM45887^T^ and *N. ferruginea* DSM43553^T^ was found to be 40.0 and 40.2%, respectively. The ANI values were in the range of 90.0 to 89.9% for these strains. Similarly, the ANIb values were 90.8% for both *N. harbinensis* and *N. ferruginea*, as determined by JSpeciesWS online services. All these values were below the threshold for bacterial species demarcation (Chun et al., 2018).

**Figure 3:**
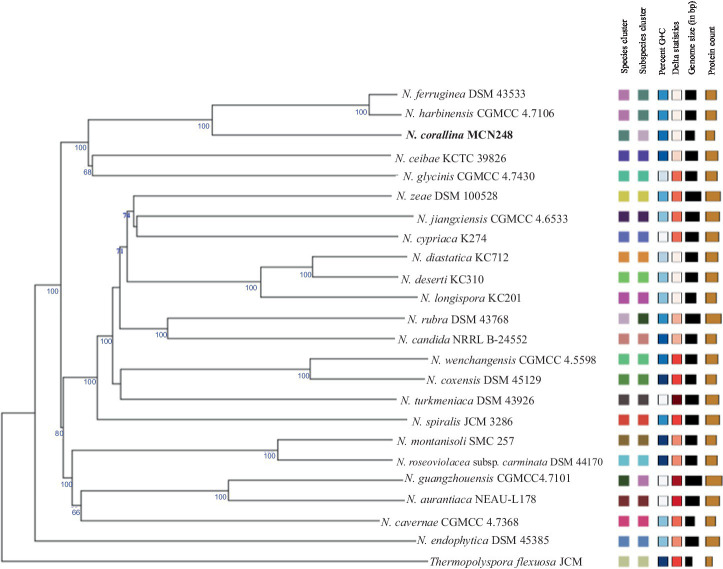
TYGS-genome blast distance phylogeny (GBDP) based on genome data of MCN248^T^, including the available genome data of *Nonomuraea* type strains in the TYGS database. *Thermopolyspora flexuosa* JCM 3056^T^ was used as the outgroup.

**Figure 4:**
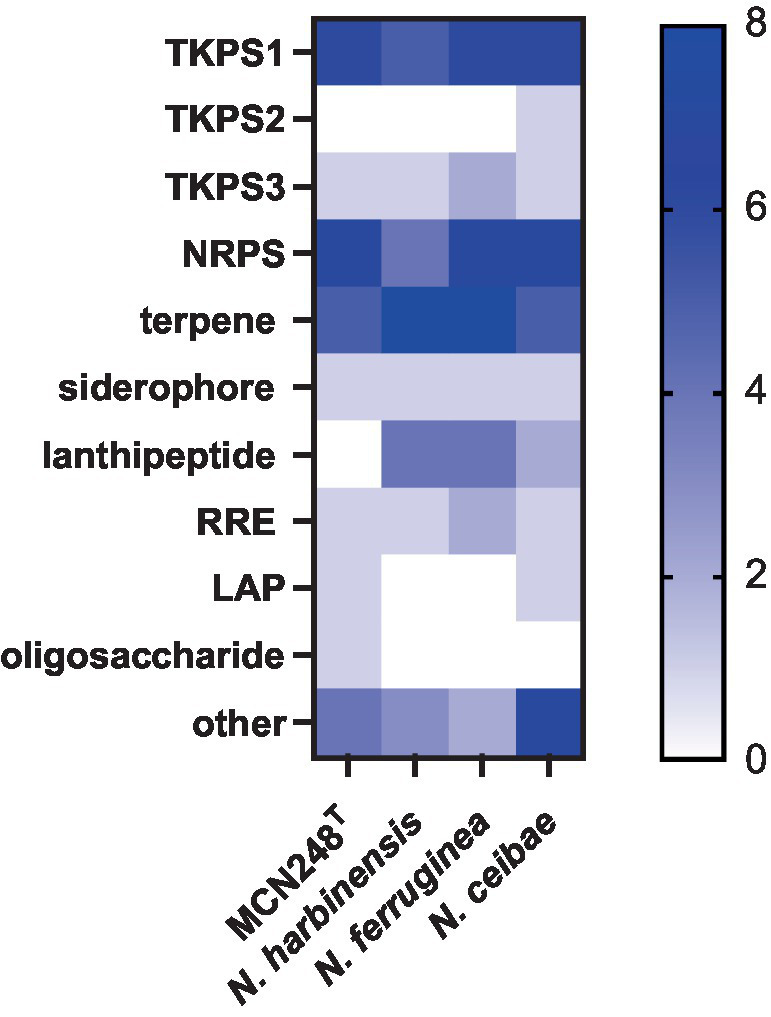
Heatmap demonstrating the numbers of biosynthetic regions in genome of strain MCN248^T^ and its closely related strains analyzed using antiSMASH software version 7.0.0beta1.

Phylogenomic tree construction based on the TYGS-genome blast distance phylogeny (GBDP) (Meier-Kolthoff and Göker, 2019) using MCN248^T^ and the available genome data of *Nonomuraea* type strains in the TYGS database supported the phylogenetic position of strain MCN248^T^ (Figure 3). Analysis with the antiSMASH tool revealed differences in the number and types of annotated biosynthetic gene clusters (BGCs) between *Nonomuraea corallina* MCN248^T^ and the closely related strains (*N. harbinensis*, *N. ferruginea*, and *N. ceibae*), as shown in the heatmap (Figure 4) and the table of numbers (Supplementary Table S2). The analyzed regions were mainly non-ribosomal peptide synthetase (NRPS), terpene, type I polyketide synthase (T1PKS), and other types. BGC numbers of MCN248^T^ obviously differ from related strains in lanthipeptide, PKS groups, and NRPS. Predicted genes involved in anticancer biosynthesis were identified in thr strain MCN248^T^ using the AntiSMASH database, highlighting its potential as a source of bioactive compounds. Notably, the gene cluster for the lipopeptides icosalide A/icosalide B showed 100% similarity, followed by the polyketide cytorhodin (37% similarity). Icosalides A and B were initially isolated from a fungal culture and reported for their cytotoxic activity against Madin-Darby canine kidney (MDCK) cells (Boros et al., 2006). Cytorhodins, belonging to the rhodomycin-antitumor compound group, were originally discovered in *Streptomyces* sp. HPL-Y11427 (Reddy et al., 1985; Hedtmann et al., 1992). These compounds have not yet been reported from *Nonomuraea* species. The discovery of these anticancer compound BGCs could lead to the discovery of new targets. For example, the novel glycopeptide A59926 was isolated from *Nonomuraea coxensis* DSM45129 based on a prior genome mining study (Yushchuk et al., 2021). Accordingly, based on a high-throughput screening of anti-colorectal cancer activity against HCT-116 cell lines, the crude extract of strain MCN248 showed partial inhibition (27.74%) at a final concentration of 50 μg/mL. This provides evidence that the strain can be further studied for the production of anticancer or other metabolites in future research endeavors.

### Chemotaxononic analyzes of strain MCN248^T^

**Figure 5:**
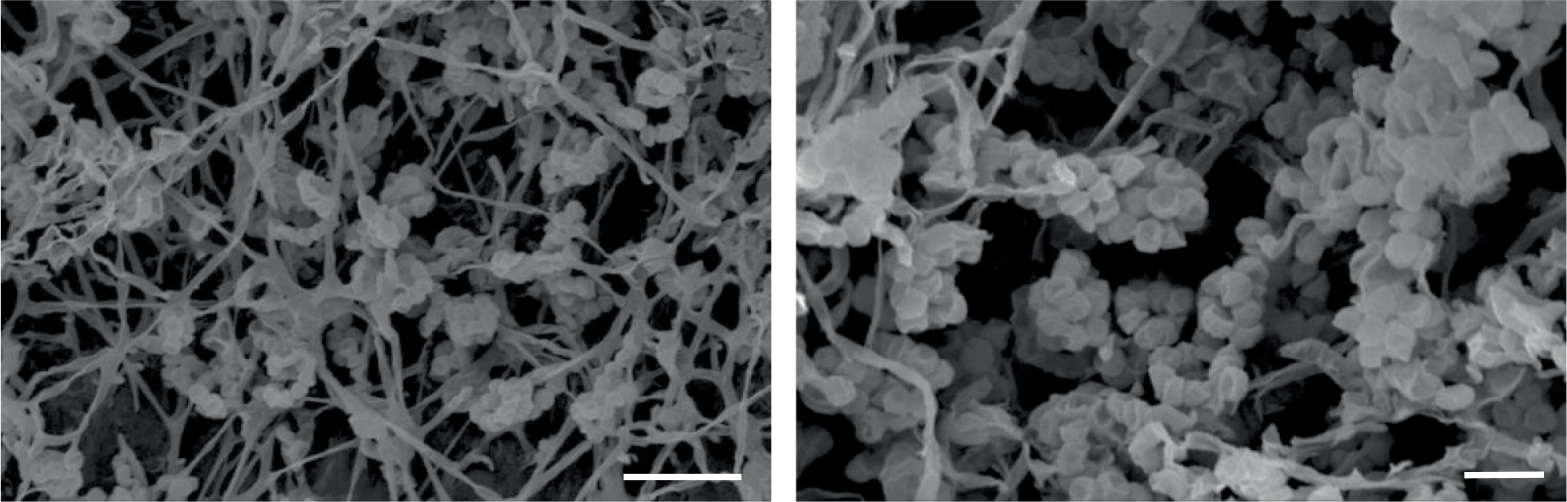
Scanning electron micrographs of spores of strain MCN248^T^ grown on ISP 3 medium for 21 days at 28°C. Bar, 5 μm (left); 2 μm (right).

Madurose, ribose, mannose, and glucose were detected as diagnostic whole-cell sugars. The type of muramic acid was acetyl, and mycolic acids were absent. The menaquinones found in strain MCN248 were MK-9(H2), with 50%, and MK-9(H4), with 44%. Phospholipids consisted of diphosphatidylglycerol, hydroxyl-phosphatidylethanolamine, phosphatidylethanolamine, phosphatidylinositol, phosphatidylglycerol, three unidentified glycolipids, two unidentified phospholipids, and two unidentified lipids (Supplementary Figure S3). The fatty acid profiles of strain MCN248^T^ were compared with those of the two reference strains, as shown in Table 1. The significant differences in the fatty acid patterns between MCN248^T^ and the closely related strains were found in the amount of iso-C16:0, iso-C15:0, and C16:0. The cellular fatty acids of strain MCN248^T^ that comprised more than 10% of total fatty acids were iso-C16:0 (40.4%), 10-methyl- C17:0 (22.0%), and C17:1ω8c (10.9%) (Table 1). The chemotaxonomic traits were consistent with those of other species in the genus (Saygin et al., 2021; Lin et al., 2022), as reported in Bergey’s Manual of Systematics of Archaea and Bacteria (Kämpfer, 2012). Madurose is the major sugar, and MK-9(H_4_), MK-9(H_2_), and MK-9(H_0_) are mainly found as the menaquinones. Iso-C16:0 and 10-methyl- C17:0 are major types of fatty acids. Phenotypic characteristic of strain MCN248T and MCN248^T^ exhibited good growth on ISP 3, ISP 4, and ISP 6. The color of the substrate mycelium varied from light coral red to burnt orange. No soluble pigment was observed in any media (see Supplementary Table S3). After 7 days of incubation, the pictures of strain MCN248 on 301 agar (24.0 g/L starch, 5.0 g/L yeast extract, 4.0 g/L CaCO_3_, 3.0 g/L peptone, 3.0 g/L meat extract, and 1.0 g/L glucose) were demonstrated in comparison with the related strains, as shown in Supplementary Figure S4. Aerial mass color was white on all the media used for cultural characterization. Vegetative mycelia were branched but not fragmented. Straight and flexuous long chain spores were produced on substrate and aerial hyphae. Sporangia were not observed. The surface of the spores was rough, and the spores were 0.5–0.6 × 0.7–1.0 μm in size (Figure 5). Growth occurred at 24–42°C, with an optimal temperature range of 28–36°C and pH of 5.0–11.0 (optimum 7–11). Strain MCN248^T^ could grow on 5% (w/v) NaCl-containing medium, while reference strains could not tolerate 5% NaCl concentration and a pH above 9.0. Several physiological traits indicated differences between the test strain and the closely related species. All degradation tests—starch hydrolysis, nitrate reduction, gelatin liquefaction, milk peptonization, and hydrogen sulfide production—yielded negative results in strain MCN248^T^, while tyrosinase activity was detected. On the other hand, *N. ferruginea* DSM43553^T^ was positive for starch hydrolysis and nitrate reduction, and *N. harbinensis* DSM45887^T^ was positive for only nitrate reduction (Table 2). The carbohydrate utilization capacity of the test strain—including D-mannitol, rhamnose, D-xylose, L-arabinose, mannose, and galactose—is the property that distinguishes it from the reference strains. Other physiological characteristics are shown in Table 2.

**Table 1:** Fatty acid compositions (%) of strain MCN248^T^ and closely related *Nonomuraea* species.

Fatty acids^a^	*N. corallina* MCN248^T^	*N. ferruginea* DSM43553^T^	*N. harbinensis* DSM45887^T^
16:0 iso	40.35	30.21	32.76
17:0 10-methyl	22.06	28.17	30.28
17:1 ω8c	10.92	6.54	5.96
16:1 iso G	4.99	2.32	4.20
17:0	4.57	3.66	4.49
14:0 iso	2.34	0.97	0.76
15:0 iso	2.22	5.41	5.25
18:0 iso	1.89	2.92	3.02
16:0	1.25	1.35	1.02
17:0 iso	0.90	0.81	1.37
18:0 10-methyl, TBSA	0.89	2.64	1.58
17:0 iso 3OH	0.88	2.62	2.56
18:1 ω9c	0.79	1.78	1.00
18:1 iso H	0.43	–	–
12:0 iso	0.34	–	–
15:1 ω6c	0.33	1.04	0.81
17:0 2OH	0.31	–	0.94
13:0	0.29	0.30	0.35
14:0	0.27	0.88	0.64
17:0 anteiso	0.17	0.60	0.55
15:0 2OH	0.16	0.28	0.34
18:1 2OH	0.14	0.34	0.42
13:0 iso	0.10	–	–

a Fatty acid contents of < 0.1% are omitted.

**Table 2:** Differential physiological and biochemical properties of strain MCN248^T^ and closely related *Nonomuraea* species.

Characteristics	1	2	3
*Colony color*	Light coral red to burnt orange	Sand to burnt orange	Pale yellow to camel
*Growth in NaCl (%, w/v)*	0–5	0–3	0–4
*Growth temperature (°C)*	24–42 (28–36)	28–38	20–34
*Growth pH*	5.0–11.0	6.8–7.8	6.0–9.0
*Biochemical test*			
Starch hydrolysis	−	+	−
Nitrate reduction	−	+	+
Utilization of carbohydrate			
D-mannitol	+	−	−
Rhamnose	+	+	−
D-xylose	+	+	−
L-arabinose	+	−	−
Mannose	+	−	−
Galactose	+	+	−
APIZYM			
Naphthol-AS-BI-phosphohydrolase	−	−	+
*β*-glucuronidase	−	−	−
*α*-mannosidase	−	+	+
*α*-fucosidase	−	−	+

Strain: 1, MCN248^T^; 2, *N. ferruginea* DSM43553^T^ [39, 40]; 3, *N. harbinensis* DSM45887^T^ [41]. Optimum temperatures are shown in parentheses. +, positive; −, negative; w, weakly positive; ND, not determined.

### Description of *Nonomuraea corallina* sp. nov.

*Nonomuraea corallina* (co.ral.li’.na. L. fem. Adj. *corallina* coral red, referring to the color of the substrate mycelium) is aerobic, gram-positive, and mesophilic actinomycetes. The colonies are light coral red to burnt orange in color. White aerial mycelium is produced, and a single spore develops on the substrate mycelia. The spores are non-motile. Vegetative mycelia are branched and not fragmented, and no sporangia are observed. The acyl type of the peptidoglycan is acetyl, and the cell wall contains meso-diaminopimelic acid. Madurose, ribose, mannose, and glucose are detected as whole-cell sugars. Major phospholipids are diphosphatidylglycerol, hydroxyl-phosphatidylethanolamine, phosphatidylethanolamine, phosphatidylinositol, phosphatidylglycerol, and unidentified lipids. The major menaquinones are MK-9(H_2_) and MK-9(H_4_), and mycolic acids are not detected. The major cellular fatty acid is iso-C_16:0_. The growth of the type strain is observed at temperatures between 24°C and 42°C, with an optimum range of 28–36°C and pH of 5–11, with an optimum pH of 7–11. The maximum NaCl concentration for growth is 3% (w/v). Strain MCN248^T^ is capable of utilizing D-mannitol, rhamnose, sucrose, D-xylose, L-arabinose, mannose, and galactose as sole carbon sources, but it shows limited growth with maltose and sorbitol. Casein hydrolysis, gelatin liquefaction, nitrate reduction, starch hydrolysis, H_2_S production, and melanin production are negative for the strain. Enzymatic activity of the API ZYM system is positive for alkaline phosphatase, α-chymotrypsin, α-glucosidase, *β*-galactosidase, *β*-glucosidase, cystine arylamidase, esterase (C4), esterase lipase (C8), leucine arylamidase, N-acetyl-*β*-glucosaminidase, trypsin, α-galactosidase, acid phosphatase, and valine arylamidase, while it is negative for lipase (C14), naphthol-AS-BI-phosphohydrolase, *β*-glucuronidase, α-mannosidase, and α-fucosidase. The G + C content of the genomic DNA is 71.7%. The type strain, MCN248^T^ (=NBRC115966^T^ = TBRC17110^T^), was isolated from Songkhla Province, Thailand. The GenBank accession numbers are OP658912 (16S rRNA gene) and JAPNNL000000000 (draft genome), respectively.

## Conclusion

Strain MCN248^T^ should be classified as a member of the genus *Nonomuraea* on the basis of both the phylogenetic analysis and chemotaxonomic characterization. However, strain MCN248^T^ was clearly distinguishable from the closely related strains by its biochemical and physiological properties (e.g., carbon utilization, starch hydrolysis, nitrate reduction, growth temperature and pH, and enzymatic activity) (Table 2). Furthermore, the dDDH and ANI values between MCN248^T^ and the closely related strains were lower than the bacterial species thresholds. Based on phenotypic and phylogenetic evidence and whole genomic data, it is proposed that strain MCN248^T^ represents a novel species of the genus *Nonomuraea*, namely, *Nonomuraea corallina* sp. nov. With a 27.74% inhibition of HCT-116 cells at a 50-μg/ml concentration of the crude extract, pure compounds may exhibit higher activity, being devoid of inactive contaminants. Nevertheless, strain MCN248^T^ holds the potential for future research on anticancer compounds or metabolite production, as it possesses biosynthetic gene clusters encoding the lipopeptides icosalide A/icosalide B (100% similarity) and the polyketide cytorhodin (37% similarity).

## Data availability statement

The datasets presented in this study can be found in online repositories. The names of the repository/repositories and accession number(s) can be found in the article/Supplementary material.

## Ethics statement

Ethical approval was not required for the studies on humans in accordance with the local legislation and institutional requirements because only commercially available established cell lines were used.

## Author contributions

CN, BI, and YI developed the ideas and designed the experimental plans. WP, AM, and CS supervised the research. CN and YI performed experiments. CN and BI analyzed the data and prepared the manuscript. All authors contributed to the article and approved the submitted version.

## Footnotes

^1^http://eztaxon-e.ezbiocloud.net
