# Supplementary material for: Nonomuraea corallina sp. nov., isolated from coastal sediment in Samila Beach, Thailand: insights into secondary metabolite synthesis as anticancer potential
Source: Front Microbiol. 2023 Nov 20;14:1226945. doi: 10.3389/fmicb.2023.1226945 (PMC10694255; doi:10.3389/fmicb.2023.1226945)
Supplement: Supplementary file 1 [file Data_Sheet_1.pdf]

Nonomuraea corallina sp. nov., isolated from coastal sediment in Samila beach, Thailand: Insights into secondary metabolite synthesis as anticancer potential

Chananan Ngamcharungchit[1, 2], Atsuko Matsumoto[3, 4], Chanwit Suriyachadkun[5], Watanalai Panbangred[6], Yuki Inahashi[3, 4*], and Bungonsiri Intra[1, 2*]

[1] *Department of Biotechnology, Faculty of Science, Mahidol University, Bangkok 10400, Thailand*

[2] *Mahidol University and Osaka Collaborative Research Center on Bioscience and Biotechnology, Bangkok 10400, Thailand*

[3] *Graduate School of Infection Control Sciences, Kitasato University, 5-9-1 Shirokane, Minato-ku, Tokyo 108-8641, Japan*

[4] *Kitasato Institute for Life Sciences (At present: Ōmura Satoshi Memorial Institute), Kitasato University, Tokyo 108-8641, Japan*

[5] *Thailand Bioresource Research Center (TBRC), National Science and Technology Development Agency, Klong Luang, Pathumthani 12120, Thailand*

[6] *Research, Innovation and Partnerships Office – RIPO (Office of the President), King Mongkut's University of Technology Thonburi, Bangkok 10140, Thailand*

*Correspondence: bungonsiri.int@mahidol.edu, y-ina@lisci.kitasato-u.ac.jp

Supplementary Figure

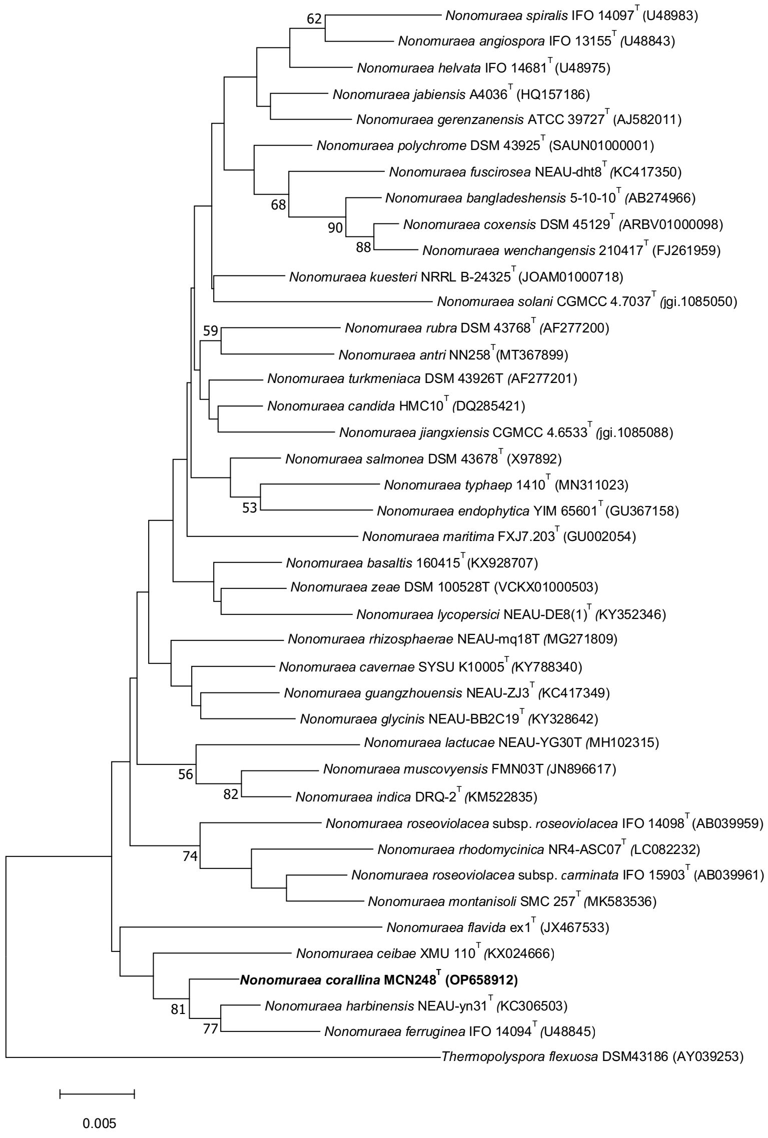

Fig. S1. The phylogenetic tree of 16S rRNA gene sequences inferred by using the Neighbour-Joining method showing the evolutionary relationships between strain MCN248^T and the closest *Nonomuraea* species.

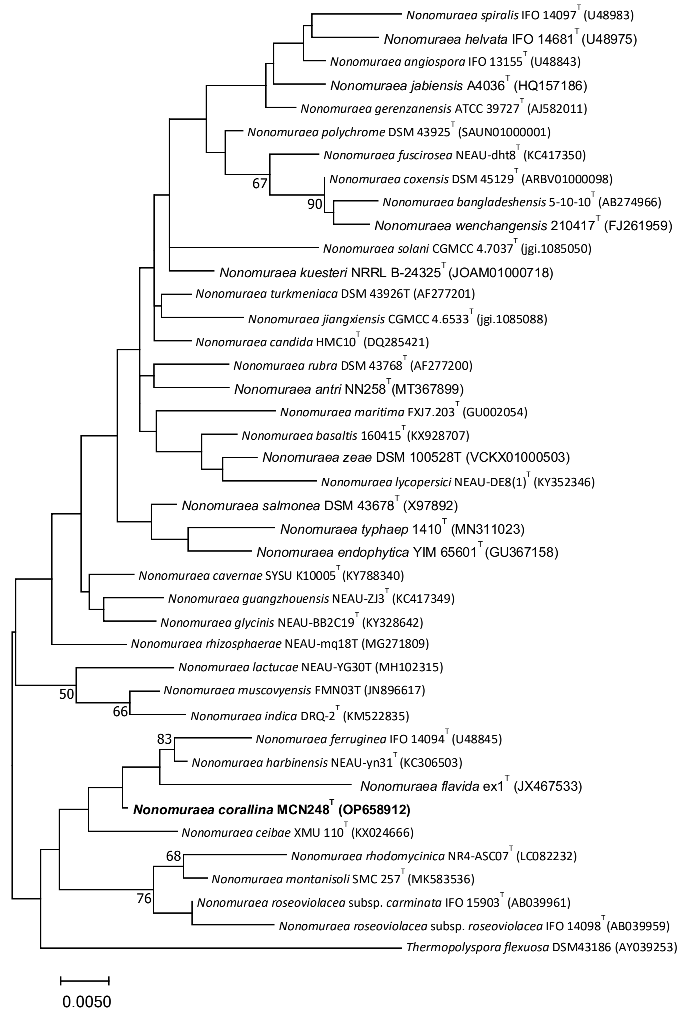

Fig. S2. The phylogenetic tree of 16S rRNA gene sequences inferred by using the Maximum Likelihood method showing the evolutionary relationships between strain MCN248^{T} and the closest *Nonomuraea* species.

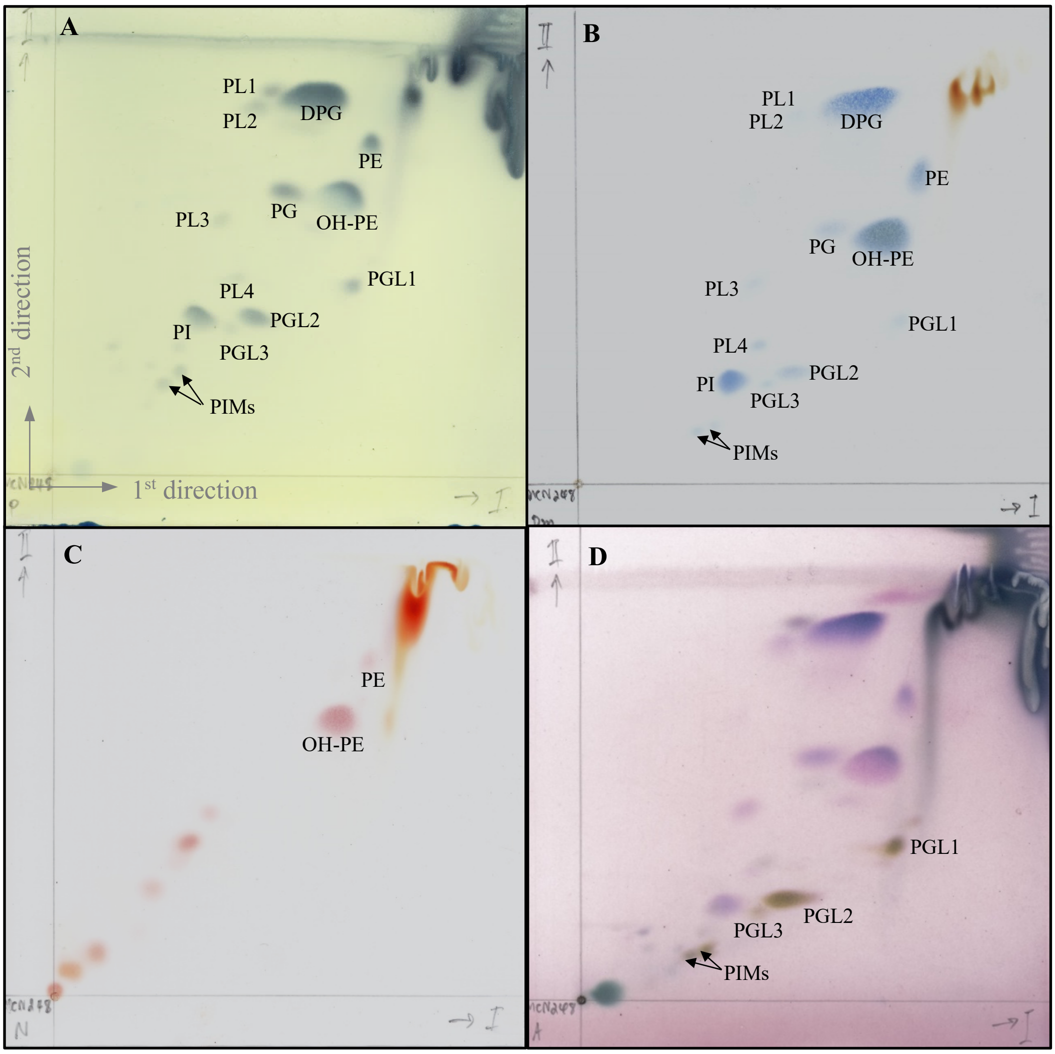

Fig. S3. Polar lipid profiles of strain MCN248^T determined by 2-dimensional thin layer chromatography, first developed using chloroform: methanol: water (32.5: 12.5: 2, v/v) with a second development using chloroform: acetic acid: methanol: water (40: 7.5: 6: 2, v/v). Lipid spots were detected using (a) molybdophosphoric acid; (b) molybdenum blue reagent; (c) ninhydrin reagent; and (d) anisaldehyde reagent. Abbreviations: DPG, diphosphatidylglycerol; OH-PE, hydroxyphosphatidylethanolamine PE, phosphatidylethanolamine; PG, phosphatidylglycerol; PI, phosphatidylinositol; PIMs, phosphatidylinositol-mannosides; PGL1, PGL2 and PGL3, unidentified phosphoglycolipid; PL1, PL2, PL3and PL4, unidentified phospholipid.

Table S1 Genome features of strain MCN248^T and its closely related reference strains.

1) *N. corallina* MCN248^T, 2) *N. ferruginea* DSM43553^T, 3) *N. harbinensis* DSM45887^T.

	1	2	3
Genome accession	JAPNNL000000000	JAPNUD000000000	JAHKRN000000000.1
Genome size (bp)	7,557,982	9,190,888	9,235,584
G+C content (%)	71.7	71.4	71.4
N50	34,089	40,735	36,598
L50	68	63	79
Number of contigs	370	371	434

Table S2 Number and type of biosynthetic gene clusters (BGCs) annotated from genome of *Nonomuraea corallina* MCN248^T and the closely related strains. The annotation was performed by using antiSMASH version 7.0.0beta1.

BGC types	*N. corallina* MCN248^T	*N. harbinensis* DSM45887^T	*N. ferruginea* DSM43553^T
TKPS1	6	7	6
TKPS2	0	0	0
TKPS3	1	2	2
NRPS	7	9	7
Terpene	5	7	8
Siderophore	1	1	1
lanthipeptide	0	2	4
RRE	1	3	2
LAP	1	0	0
oligosaccharide	1	0	0
other	4	2	2

Table S3 Cultural features of strain MCN248 ^{T} and its closely related reference strains.

1) *N. corallina* MCN248^{T}, 2) *N. ferruginea* DSM43553^{T}, 3) *N. harbinensis* DSM45887^{T},

–: Not determined.

media	1	2	3
ISP 2			
Growth	Poor	Moderate	Poor
Growth colour	Light Coral Red (6la)	Burnt Orange(5pc)	Camel(3ie)
Reverse colour	Coral (6lc)	Terra Cotta(5pe)	Camel(3ie)
Aerial mycelium	Moderate	Moderate	Abundant
Aerial mass colour	White (a)	White(a)	White(a)
Soluble pigment	none	none	none
ISP 3			
Growth	Abundant	Abundant	Abundant
Growth colour	Light Coral Red (6la)	Light Coral Rose (6ga)	Pastel Yellow (1db)
Reverse colour	Light Coral Red (6la)	Brite Coral Rose (6ia)	Pastel Yellow (1db)
Aerial mycelium	Moderate	Moderate	Poor
Aerial mass colour	White (a)	White (a)	White (a)
Soluble pigment	none	none	none
ISP 4			
Growth	Moderate	Abundant	Moderate
Growth colour	Burnt Orange (5nc)	Pearl (3ba)	Ivory (2db)
Reverse colour	Coral (6lc)	Pearl (3ba)	Ivory (2db)
Aerial mycelium	Moderate	Poor	-
Aerial mass colour	White (a)	Terra cotta (5pe)	-
Soluble pigment	None	none	none
ISP 5			
Growth	Poor	Moderate	Moderate
Growth colour	Light Coral Red (6la)	Sand (3cb)	Pearl (3ba)
Reverse colour	Light Coral Red (6la)	Sand (3cb)	Pearl (3ba)
Aerial mycelium	Moderate	Moderate	-
Aerial mass colour	White (a)	Light Coral Red (6la)	-
Soluble pigment	none	none	none
ISP 6			
Growth	Moderate	Moderate	Moderate
Growth colour	Brite Coral Red (6na)	Cinnamon (3le)	Topaz (3ne)
Reverse colour	Brite Coral Red (6na)	Cinnamon (3le)	Topaz (3ne)
Aerial mycelium	Trace	-	-
Aerial mass colour	White (a)	-	-
Soluble pigment	none	none	none
ISP 7			
Growth	Poor	Moderate	Moderate
Growth colour	Brite Coral Red (6na)	Sand (5lc)	Sand (3cb)
Reverse colour	Chinese red (6pc)	Light Coral Red (6la)	Sand (3cb)
Aerial mycelium	Moderate	Moderate	Poor
Aerial mass colour	White (a)	Shell Pink (5ba)	Pearl Pink Shell (3ca)
Soluble pigment	none	none	none

Table S4 Figures of strain MCN248^{T} and its closely related reference strains' cultural characteristics on 301 agar after 7 day-incubation.

Strains	Cultural characteristics on 301 agar	
N. corallina MCN248^{T}	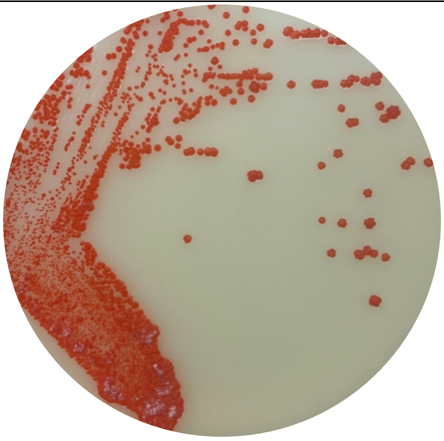	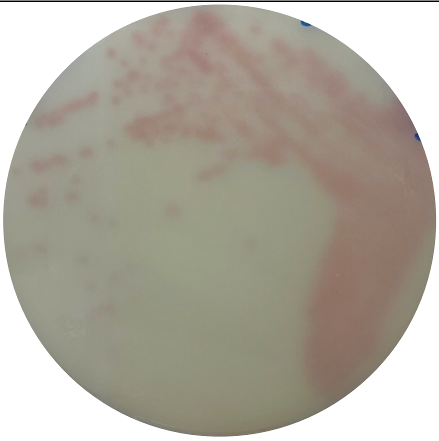
N. harbinensis DSM45887^{T}	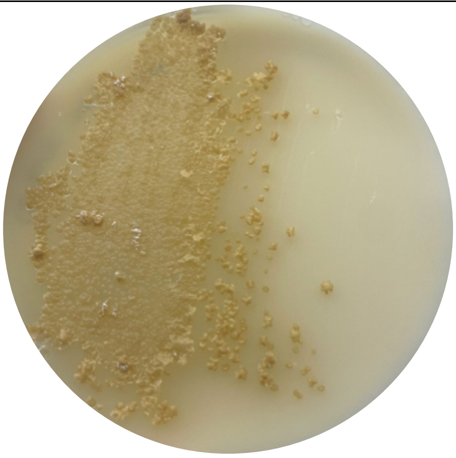	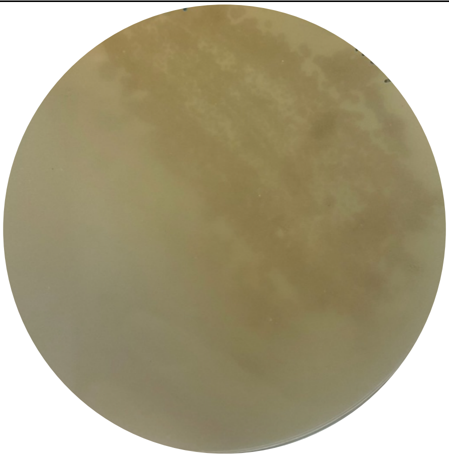
N. ferruginea DSM43553^{T}	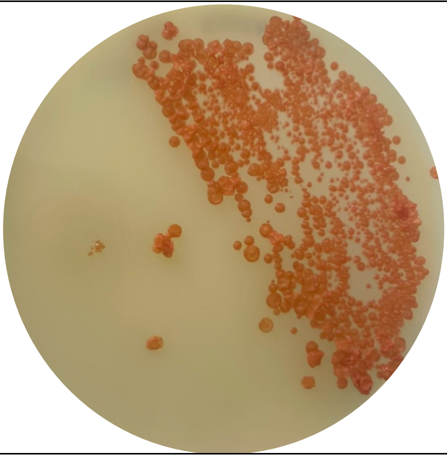	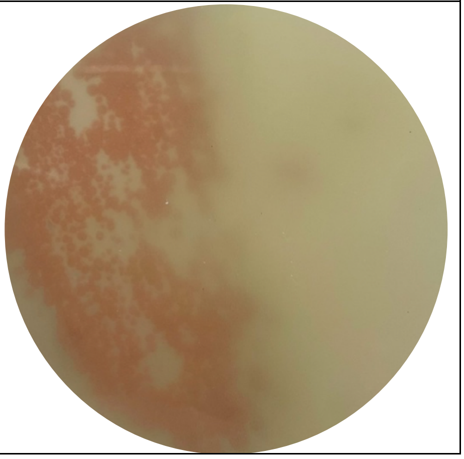
